# Quantification of beam steering with an ionization chamber array

**DOI:** 10.1002/acm2.12315

**Published:** 2018-03-25

**Authors:** Song Gao, Peter A. Balter, Benjamin Tran, Mark Rose, William E. Simon

**Affiliations:** ^1^ Department of Radiation Physics The University of Texas MD Anderson Cancer Center Houston TX USA; ^2^ Department of Physics Rice University Houston TX USA; ^3^ Sun Nuclear Corporation Melbourne FL USA

**Keywords:** beam steering, beam symmetry, ionization chamber array, quality assurance

## Abstract

Routine quality assurance for linear accelerators (linacs) usually involves verification of beam steering with a water scanning system. We established a beam steering procedure that uses a 2D ionization chamber array (ICA) and verified the equivalence of beam symmetry between the ICA and a water scanning system. The ICA calibration accuracy, reproducibility and stability were evaluated and the uncertainty in the measurement of beam symmetry due to the array calibration was examined. Forty‐five photon beams and 80 electron beams across 7 Varian C‐series and 4 TrueBeam linacs were steered in the radial and transverse directions using an ICA. After beam steering, profiles were re‐measured using the ICA and in‐water using a 3D Scanner (3DS). Beam symmetries measured with the ICA and 3DS were compared by (a) calculating the difference in point‐by‐point symmetry, (b) plotting the histogram distribution of the symmetry differences, and (c) comparing ICA and 3DS differences with their respective Varian symmetry protocol analysis. Array calibrations from five different occurrences (2012 to 2016) over six different beams reproduced within 0.5%. The uncertainty in beam symmetry was less than 0.5% due to the uncertainties in the array calibration. After all beams were steered using the ICA, the point‐by‐point symmetry differences between ICA and 3DS at the off‐axis positions of 20% and 80% of field size for all beam profiles indicated that 95% of point‐by‐point symmetry comparisons agreed within 0.7%, and 100% agreed within 1.0%; after steering with the ICA 97.8% of photon beam profiles (88 of 90) and 97.5% of electron beam profiles (156 of 160) had symmetry within 1% when measured with the 3DS. All photon and electron beam profiles had symmetry within 1.1% and 1.2%, respectively, for profiles measured with the 3DS. Our data demonstrate that a calibrated ICA can be used to steer photon and electron beams achieving beam symmetry within 1% when re‐measured with a 3D water scanning system.

## 1 INTRODUCTION

Beam steering on a clinical linear accelerator (linac) is traditionally performed with a water scanning system during annual quality assurance (QA) checks to ensure the consistency of the beam profile.^1^, ^2^, ^3^ The goal of the steering is to ensure that the beam is symmetric in both the in‐plane and cross‐plane directions. The use of a water scanning system to accomplish the beam steering process is considered the gold standard but is laborious, time consuming, and has uncertainties.^4^ We propose that beam steering can be accomplished much more efficiently with no loss in accuracy using a two‐dimensional (2D) array. A commercially available ionization chamber array (ICA) (IC PROFILER, Sun Nuclear Corp., Melbourne, FL) was used in this study. This ICA was previously demonstrated to be an effective tool for evaluating changes in photon beam energy,^5^, ^6^, ^7^ which is another important part of linac annual QA.

The metric used most often for beam symmetry is the central axis (CAX) point difference symmetry which is defined as,

(1) $$\mathrm{Sym}=\max_{i\in n}\left(\frac{D_{i}-D_{-i}}{D_{\mathrm{CAX}}}\right)\times 100\%$$

where, *D*_*i*_ and *D*_*‐i*_ are the measured profile intensities at a pair of points located on opposite sides and equal distance from the CAX along the same axis (mirror points), D_CAX_ is the intensity at the central axis, and *n* is the number of mirror points sampled within 80% of the field size. Please note, some software (e.g. 3D scanner) only reports the absolute value.

The uncertainty in determining symmetry is directly related to the uncertainty in measuring the relative intensities at mirror points. This uncertainty will have a systematic components due to the uncertainties in the corrected response of the detector(s) at each of these points and setup uncertainties in the alignment of the measurement system to the beam. There are also random components due to beam fluctuations as well as the random measurement uncertainties associated with any measurement system.

ICAs have large numbers of detectors, and the uncertainty in the calibration of the sensitivity between any pair of detectors at mirror points will result in systematic uncertainties in the symmetry measurements. Setup uncertainty can be minimized by careful setup of the array to the beam center and a metric of beam center is reported in real‐time during profile measurements based on the radiation field edge location. Further uncertainty will result from random variations that can be minimized by acquiring an integrated signal over a large number of monitor units. Evaluation of calibration accuracy is essential to ensure that symmetry measured with the ICA is sufficiently accurate to be used in beam steering.

Beam symmetry determined with a water scanning system has little or no systematic uncertainty, as the same detector is used for the mirror points. Setup uncertainty is minimized using the scanners automatic setup which determines the CAX using the edges of the radiation fields with two collimator angles 180^o^ apart. There is random uncertainty, as the beam profile can change between the times the detector is at a point and when it is at the mirror point; this is generally neglected during beam steering. There is also random uncertainty due to statistical fluctuations in the measurements at each point. These uncertainties are minimized using a reference detector during beam scanning and smoothing of the profile data before calculating the symmetry. In this work, a 3D water scanner (3DS) (3D SCANNER, Sun Nuclear Corp., Melbourne, FL, USA) was used with proper scan speed and data smoothing, for the sake of this work, we considered this the gold standard for determining symmetry.

The goal of this study was to evaluate that accurate beam steering can be achieved with the ICA instead of the 3DS. We examined the uncertainties and stability of the ICA calibration and how they affect our ability to determine beam symmetry. We developed a procedure for beam steering with the ICA and applied it to steering a large number of photon and electron beams. In each case, we scanned the profiles and re‐measured the symmetry with a 3DS for comparison. We demonstrated that we can achieve our institutional standards of symmetry within 1% when beams were steered using the ICA.

## 2 MATERIALS AND METHODS

### 2.A Array calibration

The 2D ICA used here is IC Profiler which has 251 ion chambers at an effective depth of 0.9 cm. The detectors are arranged along the four axes, in‐plane (y), cross‐plane (x), and two diagonals. The x‐ and y‐axes are 32 cm in length with 0.5 cm detector spacing except for missing detectors at ±0.5 cm off axis position in the x‐axis. The y‐ and x‐axes were aligned to correspond to in‐plane (gun‐target direction) and cross‐plane and were used for the symmetry measurements and beam steering in this work. The array calibration normalizes the relative response of the off‐axis detectors to the central axis detector. A wide‐field calibration technique 8, 9 is used to determine the correction factors of off‐axis chambers relative to the response of the central axis chamber. We created a unique calibration file for each combination of energy and build‐up, with the files stored in ASCII format. This was done multiple times over the course of this study to enable us to review short‐ and long‐term stability of the correction factors.

Array calibrations were performed according to the manufacturer's instructions. For photon beams the source to ICA surface distance (SSD) was 100 cm and the field size was 35 × 35 cm^2^. For electron beams the SSD was 110 cm and a 25 × 25 cm^2^ cone was used. An extended SSD was used to ensure that all detectors used in measuring at the nominal SSD, including in the penumbra region, are in the calibration field. Detectors outside the field are automatically excluded by the software. For both photons and electrons the field sizes used for calibration are larger than the field sizes used in subsequent measurements. Calibrations were performed on Varian C‐series and TrueBeam linear accelerators (Varian Medical Systems, Palo Alto, CA, USA) and were done for flattened beams with energies of 6, 10, 15, and 18 MV, and flattening filter free (FFF) beams with energies of 6 MV FFF, and 10 MV FFF photons and for 6, 9, 12, 16, and 20 MeV electrons. The same calibrations were used for the same nominal energies between the TrueBeam and C‐series machines. All photon calibrations were done with 2 cm of solid water on top of the ICA. Calibrations were done with no buildup for all electrons and also with 1 cm for 9 MeV and 2 cm for 12 MeV and higher. The use of additional buildup material did not affect the calibration accuracy, and thus the data with and without buildup were combined for this work.

#### 2.A.1 Reproducibility and stability of the array calibration

Array calibration reproducibility and stability were quantified by determining each detector's correction factor from multiple calibrations. To test the short‐term reproducibility of the array calibration, we performed the array calibration three times on the same day on the same linac. For each calibration, the ICA was set up independently from the previous setup and the software was restarted. To test the long‐term stability, we compared the calibrations done as part of this work to those we did on the same device under the same conditions upon initial acceptance of the device (4 yr before) and a periodic check (3 yr before). The percent error [$P\_\mathrm{erro}r_{n}\left(\mathrm{cf}\right)$] is defined 8 as:

(2) $$P\_\mathrm{erro}r_{n}\left(\mathrm{cf}\right)=\left[{\left(\frac{\mathrm{cf}}{\overset{¯}{\mathrm{cf}}}\right)}_{n}-1\right]\cdot 100\%$$

where: cf is the correction factor for a detector and $\bar{\mathrm{cf}}$ is the mean correction factor of the detector across of the three calibrations done on the same day.

#### 2.A.2 Symmetry accuracy and array calibration

To measure the reproducibility of the beam profile measurements taking into account both the beam and detector stability, we measured the same profile five times each for five photon and five electron beams. All measurements were done for photons using a 30 × 30 cm^2^ field and for electrons using a 25 × 25 cm^2^ cone; in both cases the surface of the ICA was set to be 100 cm from the source. Measured intensity (D) for each detector is the integrated signals over 200 monitor units (MU) corrected by the energy and build‐up specific array correction factors. Beam profile stability was quantified by determining the standard deviation in intensity for each detector for each beam for the 5 measurements with the ICA.

To measure the accuracy of the CAX point difference symmetry metric [Eq. (1)], we examined the combined uncertainty of the calibrations for each detector pair by measuring beam profiles an additional 5 times each but with the ICA rotated 180 degrees using the same measurement setup as for the reproducibility study. The accuracy of symmetry measurements was quantified by calculating the differences in profile intensity D at a given point in space when determined with a detector with the ICA at its standard orientation and its mirror with the ICA rotated 180° [Eq. (3)]

(3) $$C_{\sigma }\left(i\right)=\frac{D_{i}\left(0^{\circ }\right)-D_{-i}\left(180^{\circ }invert\right)}{D_{\mathrm{CAX}}}\times 100\%$$

where *D*_*i*_(0°) is the intensity measured at a given detector with the array at its standard orientation and *D*_*−i*_ (180° *invert*) is intensity measured at the mirror detector with the ICA rotated 180° and D_CAX_ is the intensity from the detector at the central axis. These intensities should be identical if the detector's corrected response is exactly the same and the beam was consistent for the integrated 200 MU used in each orientation of the ICA. In practice, it will be nonzero because of systematic uncertainty in the array calibration combined with the random uncertainties from the measurements. For each detector, the average of the 5 irradiations provides the systematic uncertainty and the standard deviation provides the random uncertainties.

The uncertainty in the measurement of beam symmetry due to the array calibration was examined by applying the random and systematic uncertainties in the array calibration to the CAX point difference symmetry equation [Eq. (4)], which is similar to Eq. (1) except that symmetry is considered on a detector‐by‐detector basis rather than the maximum point symmetry difference. The CAX point difference symmetry is

(4) $$Sym\left(i\right)=\frac{D_{i}\left(x\right)-D_{-i}\left(-x\right)}{D_{\mathrm{CAX}}}\times 100\%$$

where *Sym*(*i*) is the difference between two measurements of profile intensity at mirror points with two detectors (a given detector and its mirror). Comparing Eqs. (1) and (3), the systematic errors of symmetry measurement due to ICA calibrations can be quantified by the calibration uncertainty C_σ_. This was studied by applying the C_σ_ to an idealized perfectly symmetric profile modeled as a geometric function and to a measured profile.

### 2.B Beam steering using 2D ionization chamber array

Beam steering using the ICA was done for 45 photon beams and 80 electron beams across 7 Varian C‐series and 4 TrueBeam linacs with a source‐to‐surface distance (SSD) of 100 cm. For photons a 30 × 30 cm^2^ field was used and for electrons a 25 × 25 cm^2^ cone was used. For all photons, based on our experience, symmetry is equivalent when measured at 10 cm and near d_max_, we used 2 cm of solid water buildup on top of the device for easy setup. For some electron energies we initially used additional buildup on top of the device. We used no additional buildup for 6 MeV, 1 cm for 9 MeV, and 2 cm for ≥12 MeV. During this work we found that the additional buildup did not improve the results for electron beams and thus we started steering all electron beams with no additional buildup on the device.

Several versions of the IC Profiler software (3.2, 3.3, and 3.4) were used. The changes between these versions affected only the user interface and not the calculations. Beams were steered using “instantaneous rate” rate mode and with the symmetry point difference metric [Eq. (1)]. This approach gives real‐time feedback on the maximum difference between any pair of mirror points within the central 80% of the field. During the “learning curve” for this system, some beams were only steered within 1%, the symmetry criteria we set when beams were steered using a 3D water scanning system. Subsequently, beams were steered to achieve symmetry within 0.5% which could be achieved with the real‐time feedback available with the ICA.

Each beam was steered in both the radial and transverse axes using the “instantaneous rate” mode on the ICA and going back and forth between the 2 axes until the symmetry on both axes were acceptable. Our procedure for steering is to disable the beam steering servos, steer the beam, then re‐zero the servos to the steered beam. Once beam steering was done for each beam, a final profile was obtained with beam steering servos engaged and at the clinical dose rate, with the ICA in “total dose” mode and 200 MU being delivered.

### 2.C Profile comparison with ICA and 3DS

To evaluate the accuracy of beam steering done with the ICA, we compared the beam profiles measured with the ICA with those measured in water with the 3DS after beam steering was completed. Photon beam profiles were scanned in a 30 × 30 cm^2^ field at d_max_ and 2.9 cm depth for flattened and FFF beams, respectively. Electron beam profiles were scanned with a 25 × 25 cm^2^ cone and depths of 1.0 cm (6e), 2.0 cm (9e), and 3.0 cm (12e, 16e, 20e) per our institutional guidelines. All data from the 3DS were smoothed with a 5‐point rolling average before analysis. Profiles measured with the ICA and 3DS were evaluated with three methods.

1. Comparing the results of the “CAX point difference symmetry” [Eq. (1)] from the ICA with that from the 3DS for the same axis of the same beam. It should be noted that the ICA reports the “CAX point difference” including a sign indicating which side of the profile is higher or lower, whereas the 3DS reports only the magnitude of the “CAX point difference.” Also, the mirror point pair chosen for the symmetry may be different between any two measurements.
2. Examining the “CAX point difference symmetry” at each pair of mirror points [Eq. (4)] between the ICA and the 3DS for a sampling of profiles with matched field size, distance from the source, and effective depth. Both the ICA and the 3DS measured profiles were exported into an Excel file. Data measured with the 3DS were interpolated to 0.5 cm spacing before export. Point‐by‐point symmetry differences were calculated between the ICA and the 3DS [Eq. (5)].
  (5) $$\Delta S\left(i\right)=Sym_{i}\left(\mathrm{ICA}\right)-Sym_{i}\left(3DS\right)$$
  where Sym_*i*_(ICA) is the symmetry measured with ICA and Sym_*i*_(3DS) is the symmetry measured with 3DS at the same off‐axis position of the profile along the same axis.
3. Examining the symmetry measured in water after beam steering was done with the ICA. Over a period of 2 yr we used the ICA to steer 45 photon and 80 electron beams either at annual calibrations or at major service (most commonly monitor chamber replacement). All of these beams were subsequently re‐measured with a 3DS to verify that the symmetry was within 1% per our institutional standard. The poststeering symmetry values from the ICA were compared with the measured symmetry values from the 3DS software. Two comparison methods were used: first, the symmetry reported by the ICA compared with that by the 3DS; second, the symmetry was re‐calculated in Excel with data exported from the ICA compared with symmetry re‐calculated in Excel with data exported from the 3DS. The re‐calculated symmetry comparison was done to overcome the shortcoming of the 3DS software reporting only the magnitude and not the direction of the symmetry.

## 3 RESULTS

### 3.A 2D array calibration accuracy

#### 3.A.1 Reproducibility and stability of the 2D array calibrations

**Figure 1:**
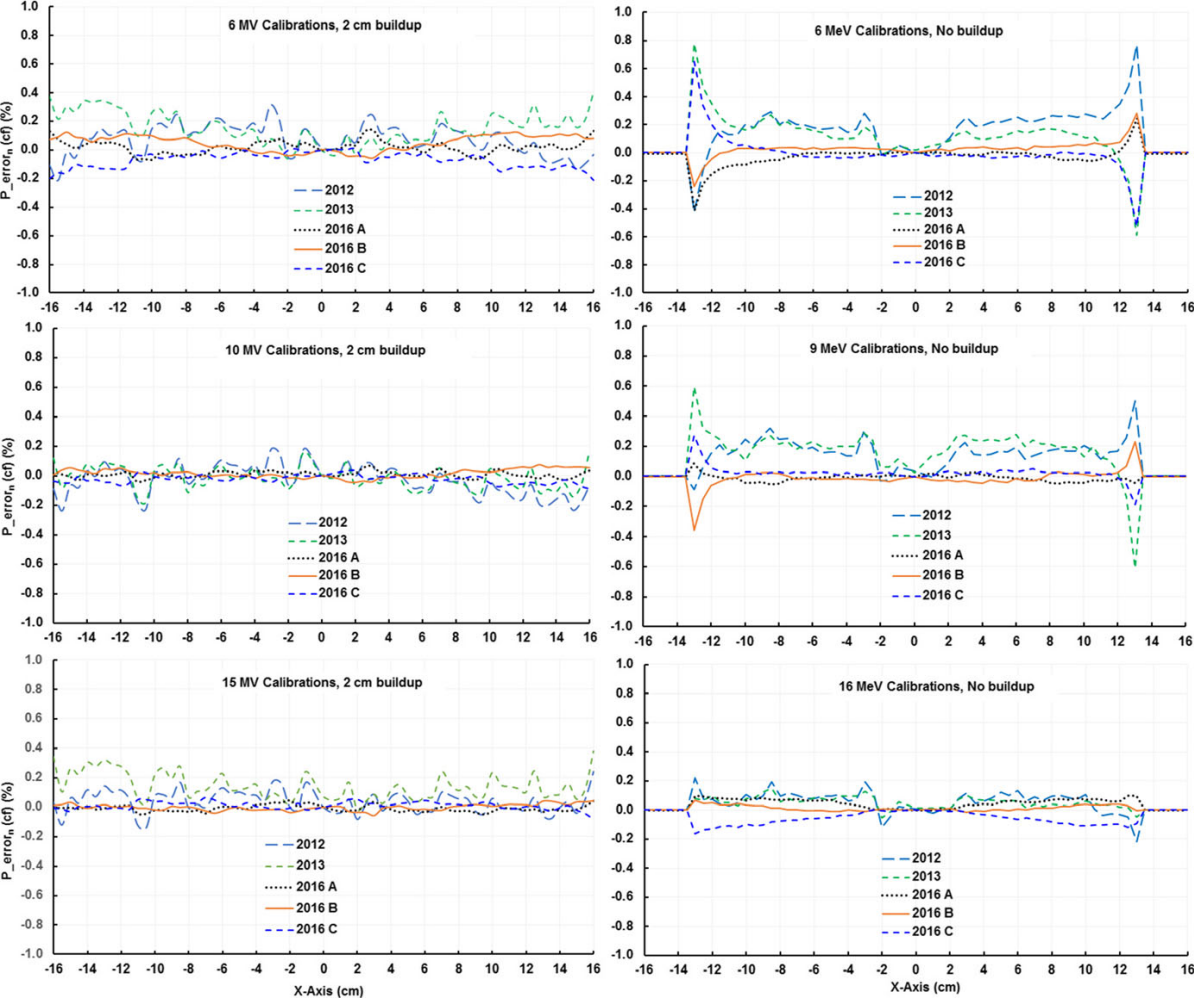
The percent errors [Eq. (2)] of detectors along *x*‐axis for array calibrations performed in the same day in 2016 and in years 2012 and 2013. Only the *x*‐axis is presented but all four axes showed similar behavior.

From repeated array calibrations during the same day in 2016, the average correction factor $\bar{\mathrm{cf}}$ for each detector was used to calculate the percent error as defined in Eq. (2). There were three calibrations from each of six beams, three photon (6‐, 10‐, and 15 MV) and three electron (6‐, 9‐, and 16 MeV). We also calculated the percent errors of the calibrations performed in 2012 and 2013 using the 2016 average factors as a reference. For both photon and electron beams, the percent errors were less than 0.5% for all detectors in the field across the five different calibrations (Fig. 1). For electron beams, the detectors located in the field edge had larger variations but were still within 0.8%, this was not observed for photon beams. No significant differences were found among the various array calibrations even though they spanned 4 yr suggesting that the device has good short‐term reproducibility and long‐term stability with respect to the array calibrations.

#### 3.A.2 Symmetry measurement accuracy with the ICA

To evaluate the ICA symmetry measurement accuracy, 5 measurements were performed with the ICA in the original position and another 5 measurements with the device rotated 180°. To determine the random portion of the uncertainty in symmetry measurements, we calculated the standard deviations (σ) of each detector for all beams in the study and used the maximum standard deviation *max*(σ) as our metric. We found the *max*(σ) to be less than 0.07% (Table 1) for all beams with electron beams having lower random uncertainty than photon beams.

**Table 1:** The maximum standard deviation (σ, %) of all detectors

Photon	6 MV	10 MV	15 MV	6x FFF	10x FFF
*max*(σ) (%)	0.05	0.07	0.05	0.03	0.03
electron	6 MeV	9 MeV	12 MeV	16 MeV	20 MeV
*max*(σ) (%)	0.02	0.02	0.02	0.01	0.02

**Figure 2:**
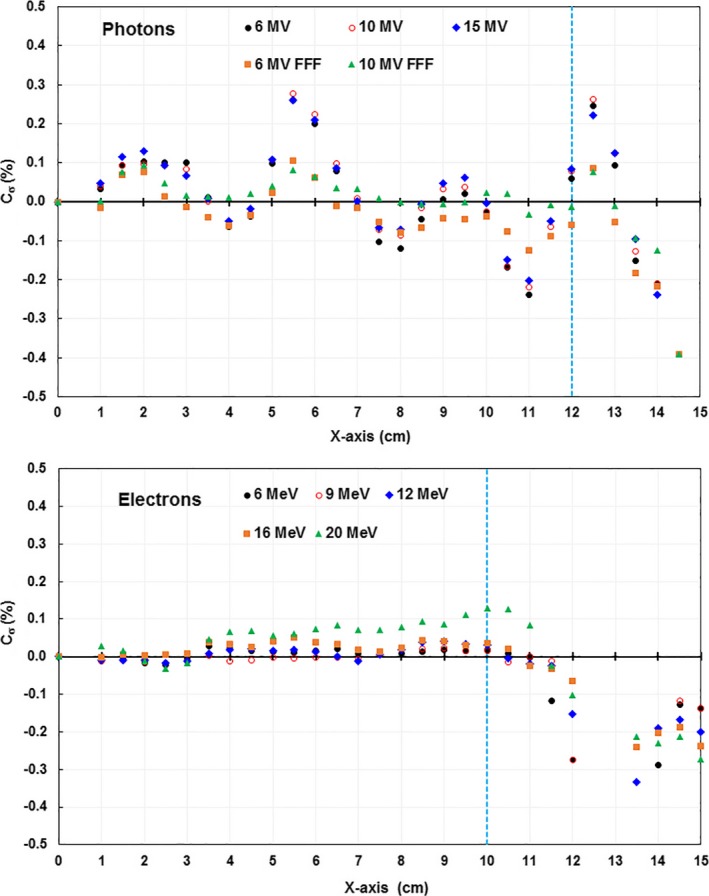
The calibration uncertainty C_σ_ of the detectors in the *x*‐axis. Data acquired at SSD = 100 cm, 30 × 30 cm^2^ field, and 2 cm solid water buildup for photons, and a 25 × 25 cm^2^ cone without buildup for electrons. The vertical blue dashed lines indicated the positions of 80% of field size.

To determine the systematic component of symmetry uncertainty due to the array calibration, we calculated the maximum calibration uncertainty *max*(C_σ_) [Eq. (3)] on the x‐ and y‐axes in each beam (Fig. 2). The *max*(C_σ_) of detectors within the central 80% of the field size for all beams was less than 0.4% (Table 2). The maximum calibration uncertainty *max*(C_σ_) with electrons, in general, shows a lower uncertainty than photons. The *max*(C_σ_) is the systematic uncertainty in the array calibration and will set an upper bound for the accuracy of symmetry measurements. The total uncertainty in symmetry measurements will be *max*(C_σ_) + $\sqrt{2}$·*max*(σ), which is the maximum systematic uncertainty combined with the random uncertainty *max*(_σ_). For all beams *max*(C_σ_) is an order of magnitude greater than *max*(σ) thus the random portion of the uncertainty can be neglected.

**Table 2:** The maximum calibration uncertainty, max(C_σ_) (%), of detectors in the x‐ and y‐axes within the central 80% of the field size

Photon	6 MV	10 MV	15 MV	6x FFF	10x FFF
*max*(C_σ_) (%)	0.37	0.32	0.27	0.18	0.22
Electron	6 MeV	9 MeV	12 MeV	16 MeV	20 MeV
*max*(C_σ_) (%)	0.09	0.09	0.10	0.18	0.29

**Figure 3:**
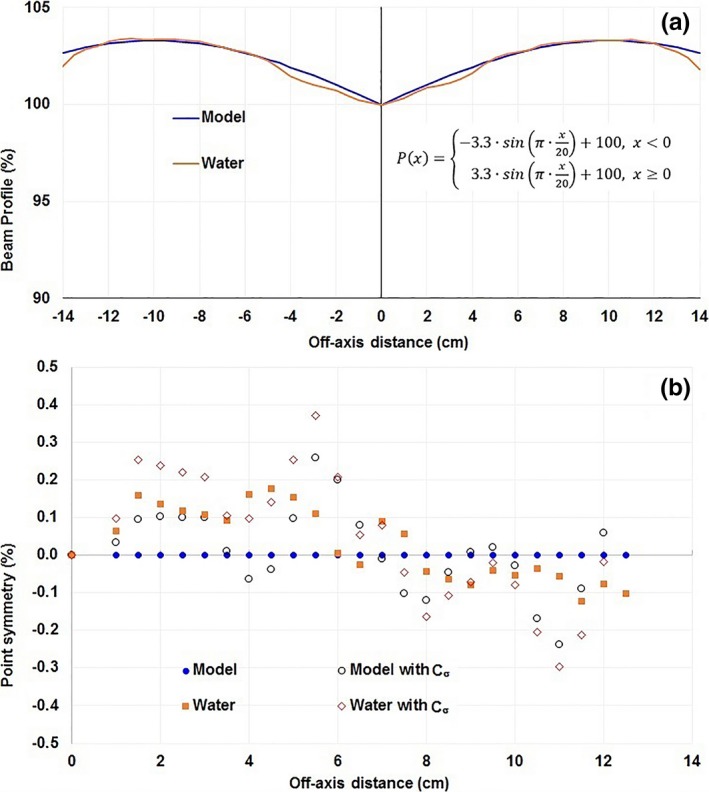
A simulation study to show the effect of array calibration uncertainty on an idealized symmetric profile (Model) and a measured in‐water profile with a 0.2% symmetry (Water) both for 30 × 30 cm^2^ fields (a). Point‐by‐point symmetry in the central 80% of these fields is plotted both with and without applying the measured uncertainty from a 6 MV ICA calibration (b).

To demonstrate that the maximum calibration uncertainty *max*(C_σ_) sets a bound for the accuracy of symmetry, we performed a simulation study by calculating the symmetry from a numerically modeled perfect beam profile and a 3DS measured beam profile that had a maximum point symmetry of 0.2% by applying the measured 6 MV ICA calibration uncertainties C_σ_. We obtained the maximum point symmetry of 0.3% for the model profile and 0.4% for the scanned profile (Fig. 3), whereas the *max*(C_σ_) of the 6 MV beam was 0.37%.

### 3.B Beam steering with the ICA

We steered 45 photon beams and 80 electron beams using the ICA and the procedures we developed although some of this data were acquired before we finalized our procedures. After the steering process was completed, we captured final profiles with the ICA. The profiles were also captured in water with the 3DS either later that same day or on the next day.

**Figure 4:**
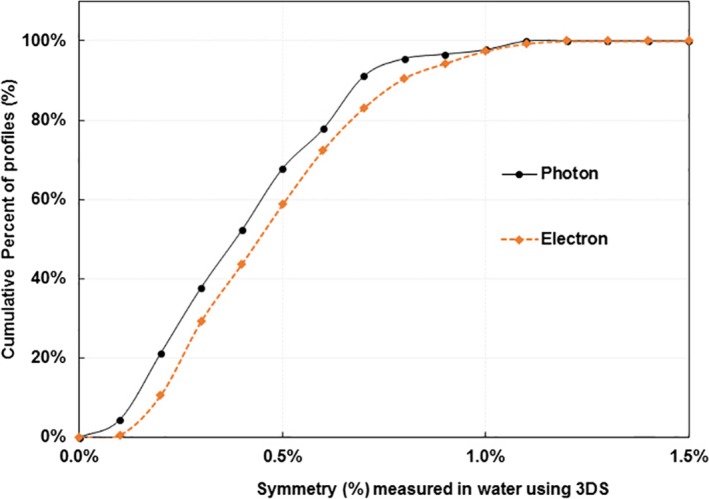
The cumulative distributions of photon beam symmetry and electron beam symmetry measured in water with the 3DS after the beams were steered with the ICA. These data represent 90 photon and 160 electron beam profiles acquired on both C‐series and TrueBeam machines.

To demonstrate how ICA based steering would work in clinical practice, we examined the probability that a beam steered using an ICA would result in a beam with a symmetry of 1.0% or less when measured using the 3DS system, for our current clinical standard. We calculated the cumulative histogram of symmetry measured using the 3DS for all photon and electron beams in this study (Fig. 4) that were steered with the ICA. The profiles measured with the 3DS showed that 97.8% of photon beam profiles (88 of 90) and 97.5% of electron beam profiles (156 of 160) had symmetry within 1%. All photon and electron beam profiles had symmetry within 1.1% and 1.2%, respectively. The beams with symmetry greater than 1% were all steered during our “learning curve” before we had set the goal for steering with the ICA to be less than 0.5% symmetry.

### 3.C Comparison of beam profiles between ICA and 3DS

Comparisons were done between the ICA and 3DS profiles in several ways: We plotted the ICA and 3DS data on the same graph for qualitative and point‐by‐point symmetry comparisons. We compared the symmetry values reported by the software for each device and also re‐calculated symmetry in Excel to have an identical metric for both devices.

**Figure 5:**
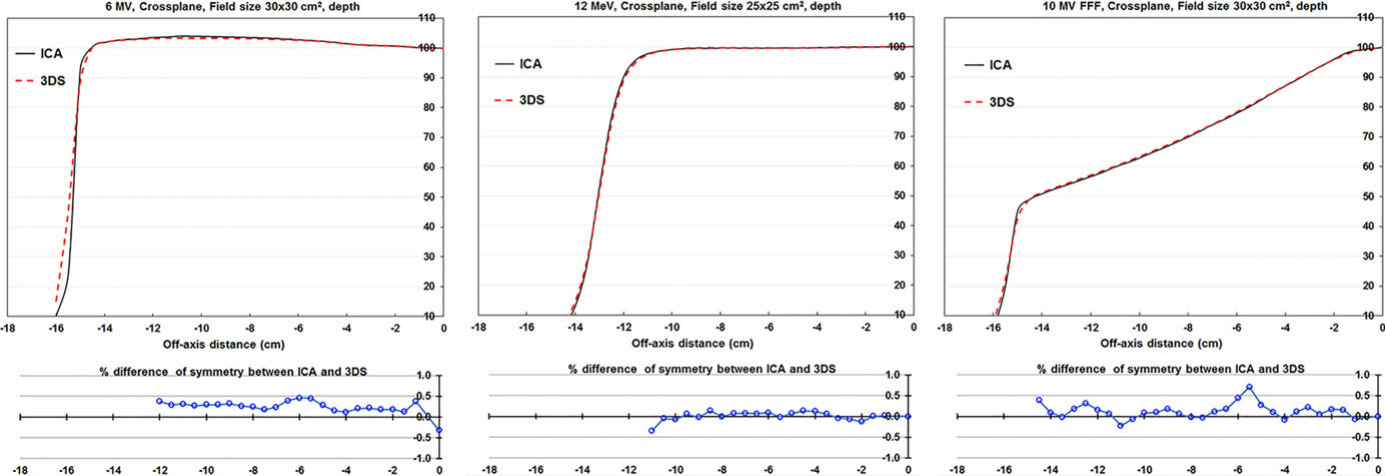
Samples of profiles measured with a 2D ionization chamber array (ICA) and 3D Scanner (3DS) (upper chart) and differences in point‐by‐point symmetry (lower chart).

The ICA and 3DS measured profiles were both exported into Excel and qualitatively compared by plotting them on the same graph and quantitatively compared by calculating the difference in point‐by‐point symmetry at each ICA detector location (Fig. 5). We found that the shape of the profiles was consistent when the two systems were used at the same SSD and the same effective depth. The differences in density between the ICA and water resulted in a 1 mm difference in source‐to‐detector distance, which was not considered significant for this analysis.

**Figure 6:**
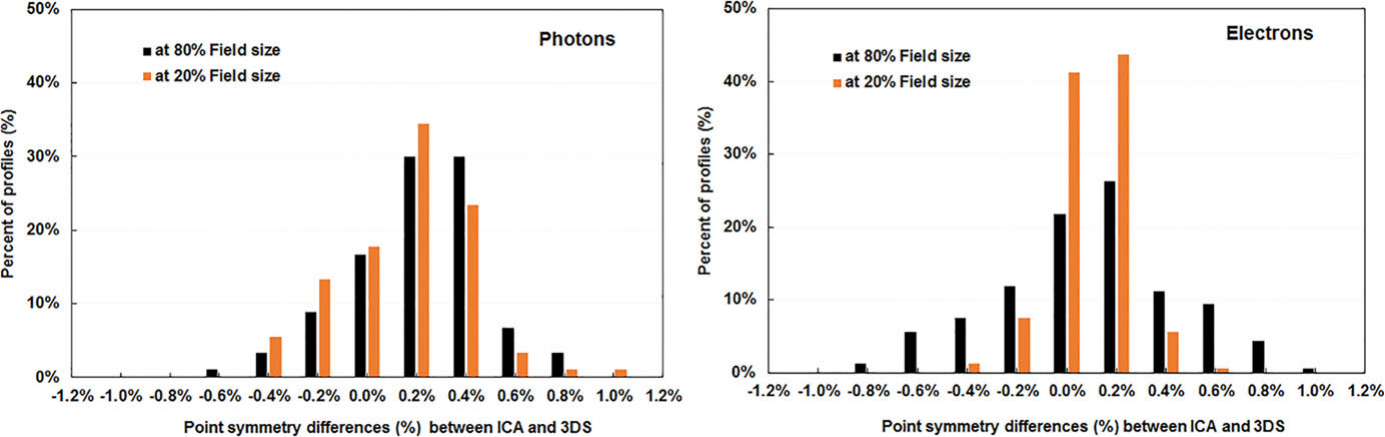
Histogram distributions of the differences of point symmetry measured with the ICA and 3DS at the off‐axis positions of 20% and 80% of field size for photon and electron beams. All beams were steered with the ICA. Differences in point symmetry are defined by Eq. (2).

We then calculated the point‐by‐point symmetry differences [Eq. (5)] between the ICA and the 3DS measured profiles at the off‐axis positions of 20% and 80% of field size. Histogram analysis of the point‐by‐point symmetry comparisons indicated that 95% agreed within 0.7% and 100% agreed within 1.0% (Fig. 6) between the two systems.

**Figure 7:**
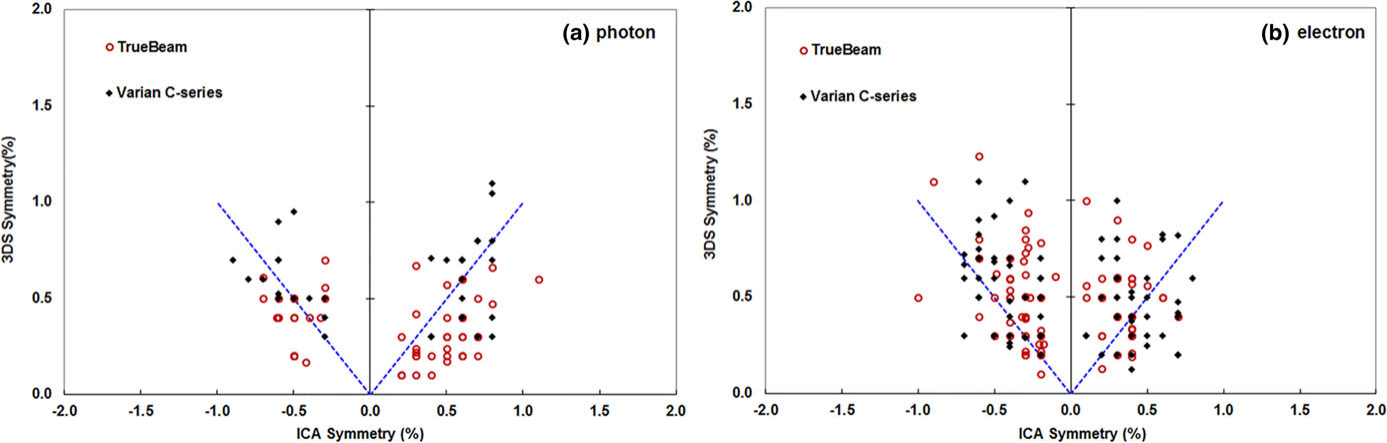
A comparison of symmetry measured and reported with the ICA vs. that measured and reported with the 3DS. The symmetry reported by the software for each system demonstrated that the ICA and 3DS use slightly different symmetry metrics even when both are set to the Varian symmetry protocol. We separated the results from Varian C‐series (black) and TrueBeam (red), and found that results did not depend on the type of machine.

**Figure 8:**
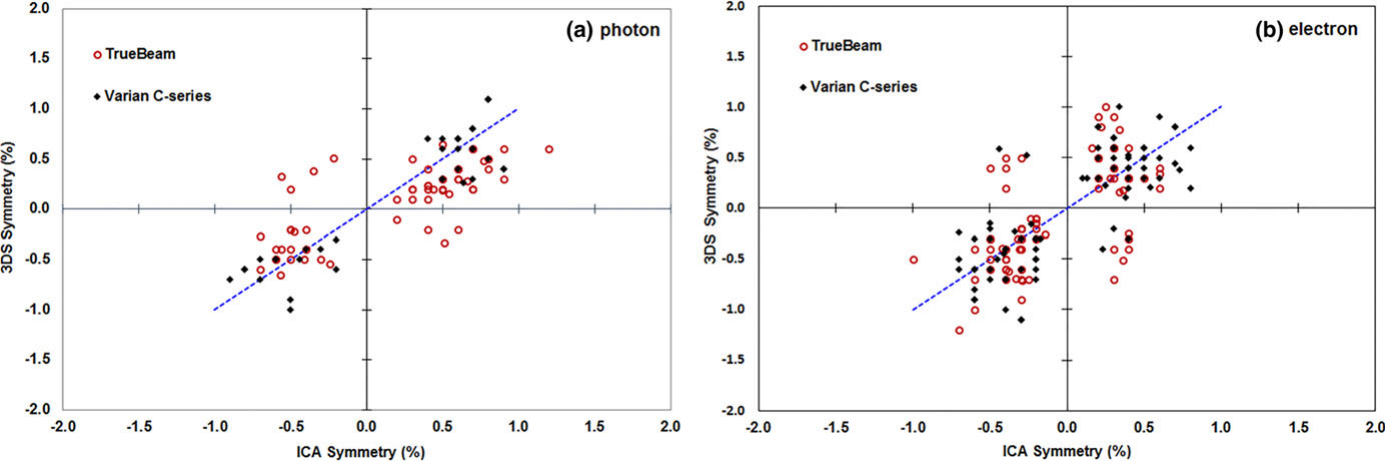
A comparison of symmetry measured with the ICA vs. that measured with the 3DS. The symmetry was recalculated in Excel to provide an identical symmetry metric between the ICA and 3DS systems. We separated the results from Varian C‐series (black) and TrueBeam (red), and found that results do not depend on the type of machine.

We then compared the symmetry measurement from the ICA software using the predefined “Varian protocol” with the value from the 3DS also using the “Varian protocol” for photon (Fig. 7a) and electron (Fig. 7b) beams. Notably, the 3DS reports only the absolute value and not the direction of symmetry, whereas the ICA reports both. To have a more complete comparison we re‐calculated the symmetry in Excel again using the “Varian protocol” to have an identical metric from ICA and 3DS systems for both photons (Fig. 8a) and electrons (Fig. 8b). We found that the two systems to be consistent at the level of 0.08% ± 0.32% for photons and 0.01% ± 0.37% for electrons using the re‐calculated symmetry data only.

## 4 DISCUSSION

The gold standard for beam steering has traditionally been a 3D water scanning system. However, a 2D detector array is far easier to setup and the real‐time feedback greatly speeds up the steering process. Our findings show that with proper calibration and procedures, beams steered with a 2D detector array can achieve the same quality in symmetry as beams steered with a 3D water scanner.

The 2D ICA used in this work had a process by which the user could recalibrate the array as well as a built‐in check procedure for the accuracy of the calibration. This array was also found to have good short‐ and long‐term stability. Physicists using other types of detector arrays will need to ensure that those detector arrays are suitable for this type of work.

We found that to effectively match the ICA measured symmetry results with those of using the 3D water scanner, we needed to:

1. Validate the array calibration to ensure that it does not introduce systematic errors;
2. Use the correct symmetry metric and understand how different software packages may report the same metric slightly different; and
3. Have the correct symmetry goal for steering such that with random and systematic errors there is a high probability that the “true” symmetry will be <1%. We recommend 0.5% or better as the goal for steering with an ICA.

The annual quality assurance on a linear accelerator is typically done with a 3D water tank, which is used for beam steering, energy checks, and profile consistency checks. Previous studies^5^, ^6^, ^7^ have demonstrated that an ICA can be used to measure changes in the energy of photon beams with a higher sensitivity than can be achieved with percentage depth dose measurements. This work can be combined with the previous studies to further reduce the need for the 3D water scanner in the annual QA process.

## 5 CONCLUSIONS

We have demonstrated that with the correct equipment and procedures, a 2D detector array can be used to steer linear accelerator photon and electron beams and achieve a resultant beam symmetry that matches that of a 3D water scanning system. However, this is true only if the array used has good calibration, the correct symmetry metric, and the correct symmetry goal during beam steering. Use of the ICA greatly speeds up the steering process because of its real‐time feedback and reduces effort by eliminating the need to setup a 3D water scanning tank.

## ACKNOWLEDGMENTS

The authors thank Christine Wogan for scientific editing of this manuscript.

## CONFLICT OF INTEREST

None.
